# The relationship between endothelial-dependent flow-mediated dilation and diastolic function in type 2 diabetes

**DOI:** 10.1007/s00592-024-02313-1

**Published:** 2024-06-07

**Authors:** Antonio Cutruzzolà, Martina Parise, Michele Cacia, Stefania Lucà, Concetta Irace, Agostino Gnasso

**Affiliations:** 1 Department of Experimental and Clinical Medicine, Magna Graecia University, Catanzaro, Italy; 2 Department of Health Sciences, Magna Graecia University, Catanzaro, Italy

**Keywords:** Endothelial dysfunction, Nitric oxide, FMD, Type 2 diabetes, Diastolic dysfunction, E/e′, Diabetic cardiomyopathy

## Abstract

**Aims:**

Diastolic dysfunction represents the earliest and most common manifestation of diabetic cardiomyopathy. Nitric oxide (NO), a potent vasodilator and anti-inflammatory mediator released from the subendocardial and coronary endothelium, favors left ventricular distensibility and relaxation. In type 2 diabetes (T2D), the NO bioavailability is reduced due to the oxidative stress and inflammatory state of the endothelium, because of chronic hyperglycemia. The aim of the present research is to evaluate the relationship between endothelial function and diastolic function in subjects with T2D.

**Method:**

Subjects with T2D and age and sex-matched healthy controls were consecutively recruited. All participants underwent flow-mediated dilation (FMD) to assess endothelial function, and echocardiography to evaluate diastolic function.

**Results:**

Thirty-five patients (6 women, 29 men) and 35 healthy controls were included in the final analysis. FMD was significantly lower in T2D than controls (4.4 ± 3.4 vs. 8.5 ± 4.3%, *p* = 0.001). T2D presented different abnormalities in diastolic function compared to controls: lower E/A (early to late diastolic transmitral flow velocity), lower septal and lateral e′ (early diastolic myocardial tissue velocity at septum and lateral wall), and higher E/e′ (surrogate of filling pressure). In subjects with T2D, we observed a significant correlation between FMD and E/e′ (r = −0.63, *p* = 0.001), lateral e′ (r = 0.44, *p* = 0.03), and septal e′ (r = 0.39, *p* = 0.05).

**Conclusions:**

Our observational study demonstrated a link between FMD and diastolic dysfunction in subjects with type 2 diabetes.

## Introduction

Diabetic cardiomyopathy is a distinct cardiac entity characterized by diastolic dysfunction, left ventricular hypertrophy, and, in a later phase, systolic dysfunction [1]. Diastolic dysfunction represents the earliest and most common manifestation of diabetic cardiomyopathy. It is characterized by impaired left ventricular (LV) relaxation at first, and subsequently by the increase of filling pressures, which can be estimated by measuring E/e′ during echocardiography [2]. The increase in E/e′ reflects a reduced LV compliance, and it predisposes to the development of heart failure with preserved ejection fraction (HFpEF) [3]. Among the numerous mechanisms proposed to explain the increase in diastolic filling pressure, endothelial dysfunction could play an important role. Under physiological conditions, nitric oxide (NO), released from the subendocardial and coronary endothelium, regulates LV distensibility and favors LV relaxation, then contributing to the whole diastolic function [4]. In diabetes, NO bioavailability is reduced due to the increased oxidative stress and inflammatory state within the vascular endothelium, as a consequence of chronic hyperglycemia [5]. One of the most used techniques to study endothelial function in vivo is the flow-mediated dilation (FMD), which exploits the ability of endothelial cells to respond to stimuli as the increased blood flow velocity, thus enhancing the release of NO and, consequently, inducing vasodilation [6]. While the relationship between diabetes and cardiovascular disease is well-established, the intricate interplay between diabetes and diastolic dysfunction remains unclear, with endothelial dysfunction configuring as a possible link in this association.

The aim of the present research is to evaluate the relationship between endothelial function evaluated by FMD and diastolic dysfunction in subjects who suffer from type 2 diabetes.

## Materials and methods

This observational study was conducted from 2020 to 2022. The research was submitted to the local Ethical Committee ‘Regione Calabria Area Centro’ and performed in accordance with the ethical standards laid down in the Declaration of Helsinki. All participants gave their informed consent prior to their inclusion in the study. Subjects with type 2 diabetes (T2D) and age and sex-matched healthy controls were consecutively recruited at the Center of Diabetes Care of the Teaching Hospital University Magna Graecia and among hospital staff. Both T2D and controls underwent FMD evaluation and complete trans-thoracic echocardiography. Inclusion criteria were: diagnosis of type 2 diabetes, age > 18 years, disease duration > 1 year, stable treatment in the last three months, and signed informed consent. Exclusion criteria were: established cardiovascular disease (previous myocardial infarction or coronary revascularization, heart failure, history of stroke, lower extremities artery disease, previous carotid stent or stenosis > 50%), history of moderate to severe valvular diseases; any active tumor; chronic kidney disease or severe liver dysfunction. Clinical information, comorbidities, diabetes complications, and ongoing treatment were collected from the electronic health records. Hypertension was defined as systolic/diastolic blood pressure ≥ 140/90 mmHg and/or the use of antihypertensive agents. Dyslipidemia was defined for total cholesterol ≥ 200 mg/dl and/or the use of statins. Microvascular complications were assigned if any of retinopathy, neuropathy or nephropathy was present in the history of the subjects. Body weight, waist circumference (WC), height, heart rate (HR), systolic (SBP), and diastolic (DBP) blood pressure were measured in all participants. Body Mass Index (BMI) was calculated using the formula weight (kg)/height (m^2^). Fasting plasma glucose (FPG) and Glycated Hemoglobin (HbA1c) were measured at the Hospital laboratory on the day of the vascular study.

### Flow-mediated dilation (FMD)

The FMD technique was used to evaluate endothelium-dependent brachial artery dilation. The study was performed by using the Philips Affiniti 70 G ultrasound system, equipped with a linear (5–12 MHz) high-resolution probe, in the morning after the patient rested in a temperature-controlled laboratory for 10 min in the supine position (22 ± 1 °C). Subjects were invited to abstain from exercise, alcohol, caffeine, food, and smoking for 12 h before the study. FMD was automatically assessed by the FMD Studio software [7], directly connected to the ultrasound machine. The technique for assessing brachial artery flow-mediated dilation (FMD) has been described in detail elsewhere [8]. Briefly, after the detection of the brachial artery, 2–3 cm above the elbow, its internal diameter (ID) was continuously measured by the automatic tracking system of the FMD Studio software. The software also automatically measured the mean brachial artery blood velocities (BV) and the baseline shear rate (SR), the SR peak, the area under the curve of SR to peak dilation (SR_AUC 0-pd_). The FMD protocol consisted of a 1-min recording of baseline brachial artery ID, BV and SR, followed by 5-min recording of ischemia, induced by a pneumatic cuff placed around the forearm and inflated at supra-systolic pressure (250 mmHg), and 3-min recording after cuff deflation to detect the reactive hyperemia (RH), which corresponds to the maximal increase of BV occurring after the release of the cuff, and the brachial artery dilation [6]. The time to peak SR and the time to peak FMD were manually calculated from the data report. The absolute change in diameter was determined as brachial artery peak diameter-baseline diameter and expressed in mm.

### Cardiac assessment

Transthoracic echocardiography was performed using the echo-doppler Philips Affiniti 70 G, equipped with a sector array (1–5 MHz) probe and simultaneous ECG recording. Linear internal measurements (interventricular septum and posterior wall thickness, end-diastolic internal diameter) of the LV were acquired in the parasternal long-axis view at the level of the mitral valve leaflet tips to obtain LV mass and relative wall thickness (RWT). Chamber size and function were assessed according to the latest guidelines [9]. Values were adjusted for body surface area (BSA). Left atrium (LA) volume was obtained from dedicated four- and two-chamber views and indexed for BSA. End-systolic and end-diastolic volumes of LV were measured by tracing the edge of ventricular walls in the apical four- and two-chamber views. Ejection Fraction (EF) was then calculated using the traditional Simpson method. Pulsed-Wave (PW) Doppler was used with a sample size of 1–3 mm settled between mitral leaflet tips in the apical four-chamber view to obtain the following variables: peak E-wave and peak A-wave velocity, mitral valve (MV) E/A ratio and MV deceleration time (DT). Tissue Doppler imaging (TDI) was applied in the same window with PW Doppler sample volume at the lateral wall and basal septum to obtain septal and lateral e′ velocity, respectively. The average E/e′ ratio was then calculated. Using continuous wave (CW) Doppler and placing the sample volume in LV outflow tract to simultaneously display the end of aortic ejection and the onset of mitral inflow, a five-chamber view was obtained to measure isovolumic relaxation time (IVRT). Tricuspidal regurgitation (TR) jet velocity was obtained with CW Doppler aligned with the color flow imaging. Right ventricular function was assessed by measuring TAPSE (tricuspid annular plane systolic excursion) with M-mode and peak systolic velocity of tricuspid annulus with TDI (S′).

### Statistical analysis

Normal distribution was assessed graphically and with the Shapiro–Wilk test. Continuous variables have been expressed as mean and standard deviation if normally distributed; otherwise, as median and interquartile range. We computed a paired t-test or Wilcoxon test to compare variables between subjects with diabetes and control subjects, as appropriate. Categorical variables were expressed as numbers and percentages, and comparisons were performed using the Chi-square test or Fisher exact test, as appropriate. Pearson correlation analyses were computed to establish the possible association between FMD and E/e′, lateral e′ and septal e′, respectively. Partial correlation analyses were used to control for the possible effect of the therapy (e.g. statin, SGLT2-inhibitors) or for the influence of baseline brachial artery ID. A linear regression model was set to establish the association between E/e′ (dependent variable) and FMD (independent variable). An adjusted model (multiple regression model) considering other possible factors influencing the diastolic function was computed to establish the independent predictive role of FMD on diastolic dysfunction. A statistical significance was considered for a *p* value less than 0.05. Data analysis was performed using IBM SPSS Statistics for Windows, Version 25.0. Armonk, NY: IBM Corp.

### Sample size calculation

A sample size calculation for one correlation with a power of 0.80 (β) and an α error of 0.05 was conducted [10] by expecting a moderate correlation between FMD and E/e′ (r = 0.6). Assuming a correlation value (r) of 0 for the null hypothesis, the sample size was estimated in 30 subjects per group to demonstrate an alternative hypothesis (r = 0.6) for a two-tailed test.

## Results

Forty subjects with T2D were consecutively enrolled. Five of them were excluded from the analysis because of the detection of unknown moderate and severe valvular disease during the echocardiographic study. Thirty-five patients (6 women, 29 men) and 35 healthy controls matched for age and sex were included in the final analysis. Subjects with diabetes had significantly more hypertension and dyslipidemia compared to controls, while BMI and cigarette habits were comparable. HR was significantly higher in T2D. The HbA1c value suggested an acceptable glycemic control. Eight (23%) subjects with diabetes had microvascular complications, in detail mild non-proliferative retinopathy. The majority of T2D subjects were treated with oral hypoglycemic agents alone or in combination with insulin (90% metformin, 17% GLP-1 analogs, 17% SGLT2 inhibitors, 34% insulin therapy). 18 (51%) were taking statins and ACE-i/ARB, while 8 (23%) were on antiplatelet and beta-blockers therapy (Table 1).

**Table 1:** Clinical and anthropometrical characteristics of subjects with type 2 diabetes and controls

	Type 2 diabetes (n 35)	Controls (n 35)	p
Age, years	58 ± 6	58 ± 6	1
Female sex, %	16	16	1
BMI, kg/m^2^	30 ± 5	29 ± 4	0.32
SBP, mmHg	130 ± 9	123 ± 11	**0.02**
DBP, mmHg	80 ± 7	78 ± 7	0.19
HR, bpm	72 ± 6	65 ± 8	0.002
Waist, cm	106 ± 12	103 ± 11	0.33
Smoke, %	28	16	0.31
Disease duration, years	10 ± 7	–	–
HbA1c, %	7.0 ± 0.9	5.2 ± 0.3	< 0.001
HbA1c, mmol/mol	53 ± 10	33 ± 4	< 0.001
FPG, mg/dl	136 ± 35	91 ± 8	< 0.001
Microvascular complications, n (%)	8 (23%)	–	–
Hypertension, n (%)	21 (60%)	11 (32%)	0.05
Dyslipidemia, n (%)	21 (60%)	8 (23%)	0.01
Metformin, %	90	–	–
GLP1-analogs, %	17	–	–
SGLT2-inhibitors, %	17	–	–
Insulin therapy, %	34	–	–
Statins, %	51	–	–
ACE-i/ARB, %	51	–	–
Antiplatelet therapy, %	23	–	–
Beta-blockers, %	23	–	–

Data are expressed as mean ± SD for continuous variables and percentages for categorical variables. *BMI* body mass index, *SBP* systolic blood pressure, *DBP* diastolic blood pressure, *HR* heart rate, *FPG* fasting plasma glucose

**Fig. 1:**
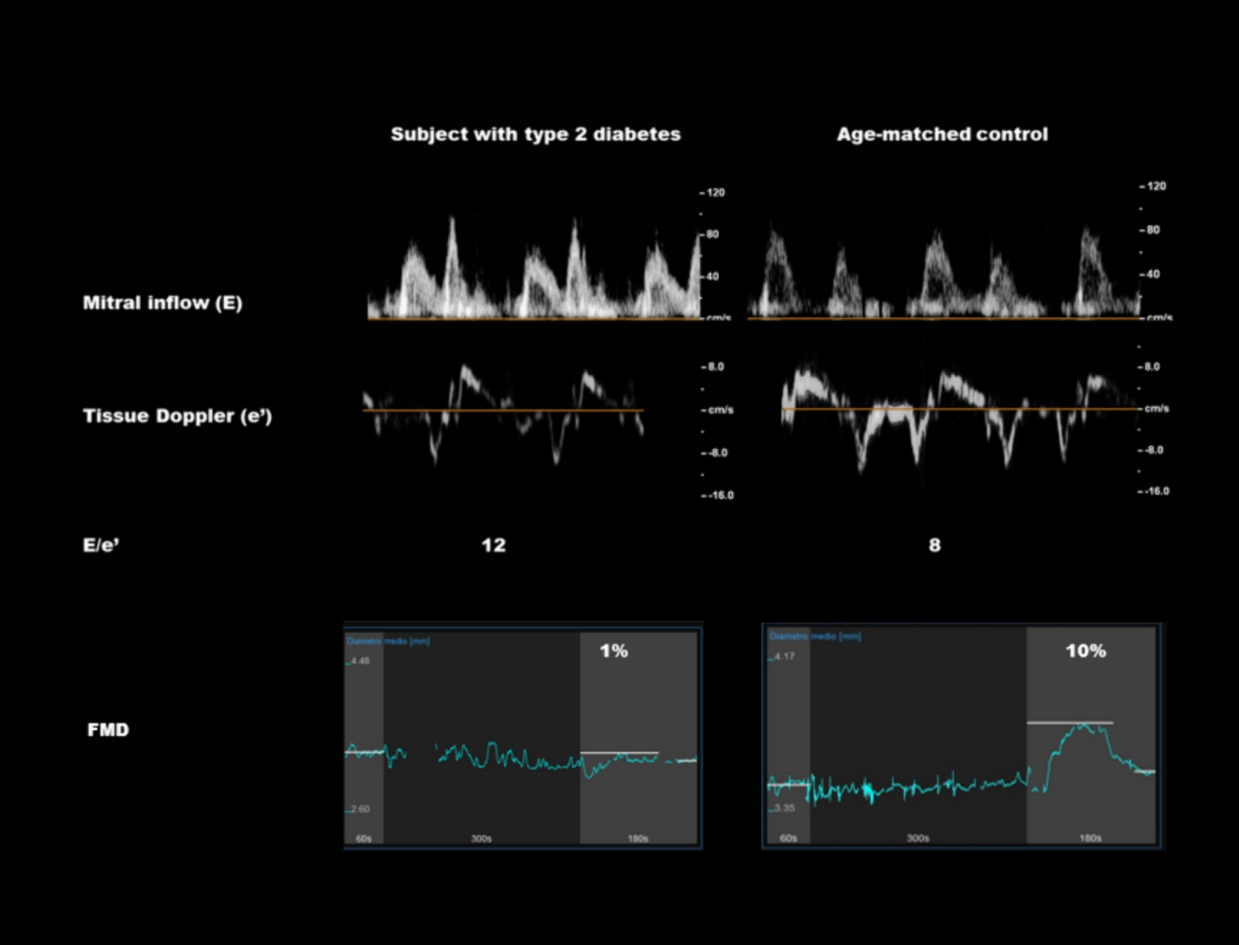
Example of diastolic function and FMD in a subject with type 2 diabetes versus an age-matched control

FMD was significantly lower in subjects with diabetes than controls, as well as the absolute change in diameter after hyperemia (Table 2). T2D also had a larger baseline and peak brachial artery diameter, and a higher SR_AUC 0-pd_ after deflating the cuff than controls (Table 2, Fig. 1). No differences in FMD were found between T2D in therapy with and without SGLT2-inhibitors.

**Table 2:** Characteristics of subjects with type 2 diabetes and controls at the flow-mediated dilation study

	Type 2 diabetes (n 35)	Controls (n 35)	p
FMD, %	4.4 ± 3.4	8.5 ± 4.3	0.001
Baseline BA diameter, mm	4.17 ± 0.60	3.66 ± 0.69	0.001
Peak diameter, mm	4.34 ± 0.57	3.96 ± 0.72	0.004
Change of diameter from baseline, mm	0.18 ± 0.16	0.30 ± 0.15	0.007
Time to peak FMD, s	52 ± 25	50 ± 14	0.68
SR_Baseline_, s^−1^ × 10^3^	0.37 ± 0.14	0.40 ± 0.17	0.43
SR_peak_, s^−1^ × 10^3^	1.11 ± 0.41	1.05 ± 0.44	0.27
SR_AUC 0-pd_, s^−1^ × 10^3^	50.0 ± 38.3	29.7 ± 26.0	0.05
Time to peak shear rate, s	12 ± 2	14 ± 2	0.008
FMD, %	4.4 ± 3.4	8.5 ± 4.3	0.001
Time to peak FMD, s	52 ± 25	50 ± 14	0.68

Data are expressed as mean ± SD for continuous variables*. FMD* flow mediated-dilation, *BA* brachial artery, *SR* shear rate, *AUC 0-pd* area under the curve from 0 (the start of hyperemia) to peak FMD

Echocardiography revealed a different LV geometry and mass between T2D and controls: T2D had higher indexed LV end-diastolic volume, interventricular septum wall, and left ventricular mass (absolute and indexed). Regarding diastolic function, E/A was lower in T2D than controls, as well as isovolumetric relaxation time (IVRT), deceleration time (DT), septal and lateral e′. E/e′ was significantly higher compared to controls (Fig. 1, Table 3). No differences were found in the left and right systolic function between T2D and controls.

**Table 3:** Echocardiographic characteristics of subjects with type 2 diabetes and controls

	Type 2 diabetes (n 35)	Controls (n 35)	p
EF, %	60 ± 4	59 ± 4	0.11
LV EDV, ml	107 ± 21	96 ± 23	0.07
LV EDVi, ml/m^2^	53 ± 8	48 ± 8	0.017
LV ESV, ml	42 ± 8	40 ± 12	0.42
LV EDSi, ml/m^2^	21 ± 3	20 ± 5	0.205
IVS, mm	11.5 ± 1.7	10.2 ± 1.5	0.007
PW, mm	9.7 ± 2.3	9.4 ± 1.5	0.61
LVEDD, mm	46 ± 5	46 ± 4	0.90
LVM, g	178 ± 43	158 ± 43	0.057
LVMi, g/m^2^	89 ± 18	79 ± 17	0.02
RWT	0.45 ± 0.09	0.41 ± 0.06	0.10
LAV, ml	51 ± 18	50 ± 16	0.80
LAVi, ml/m^2^	27 ± 8	24 ± 6	0.15
E vel, cm/s	74 ± 20	75 ± 16	0.45
A vel, cm/s	83 ± 13	75 ± 17	0.038
E/A	0.9(0.3)	1.0(0.3)	0.015
IVRT, ms	73 ± 20	87 ± 18	0.027
DT, ms	196 ± 35	228 ± 66	0.018
Septal e′, cm/s	6.9 ± 1.8	8.5 ± 1.8	0.001
Lateral e′, cm/s	9.6 ± 2.3	10.9 ± 2.7	0.05
E/e′	9.5 ± 2.2	8.1 ± 1.5	0.01
TR vel, cm/s	2.4 ± 0.3	2.4 ± 0.3	0.89
TAPSE, mm	24 ± 4	25 ± 3	0.66
S′ RV, cm/s	12 ± 2	13 ± 3	0.33

Data are expressed as mean ± SD for continuous variables and median (IQR), as appropriate*. EF* ejection fraction, *LV* left ventricle*, EDV* end-diastolic volume, *EDVi* end-diastolic volume index, *ESV* end-systolic volume, *ESVi* end-systolic volume index*, IVS* inter-ventricular septum*, PP* posterior wall, *LVEDD* left ventricular end-diastolic diameter, *LVM* left ventricular mass, *LVMi* left ventricular mass index, *RWT* relative wall thickness, *LAV* left atrium volume, *LAVi* left atrium volume index, *IVRT* iso-ventricular relaxation time, *DT* deceleration time, *TR* tricuspid regurgitation, *TAPSE* tricuspid annular plane systolic excursion, *RV* right ventricle

**Fig. 2:**
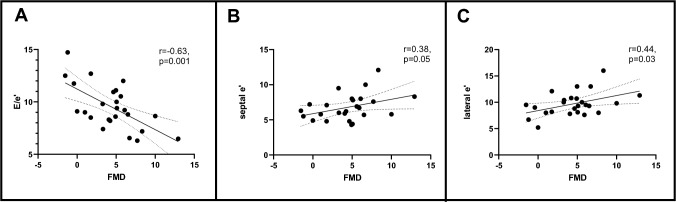
Scatterplot of the correlation between FMD and E/e′ (**A**), septal e′ (**B**), lateral e′ (**C**) in subjects with type 2 diabetes

In subjects with T2D, we observed a significant inverse correlation between E/e′ and FMD (r = −0.63, *p* = 0.001) (Fig. 2). The higher the E/e′ ratio, the lower the FMD. Moreover, FMD positively correlated with lateral e′ (r = 0.44, *p* = 0.03), and septal e′ (r = 0.38, *p* = 0.05) (Fig. 2). These correlations remained significant while controlling for the effect of statin or SGLT-2 inhibitors therapy or while controlling for the baseline BA diameter.

No correlations were found between FMD and, respectively, E/e′ (r = −0.13, *p* = 0.53), lateral e′ (r = −0.02, *p* = 0.92) and septal e′ (r = 0.33, *p* = 0.11) in controls.

In a linear regression model with E/e′ ratio set as the dependent variable and FMD as the independent variable, FMD explained 39.5% of the variability of E/e′ (R square 0.395, F = 15, *p* = 0.001). The regression formula to predict E/e′ ratio was: E/e′ = b0 + (b1*FMD) = 11.2 + (−0.39*FMD). FMD remained a significant independent predictor for E/e′ (model data: R square 0.79, *p* = 0.02, FMD beta = −0.30, standardized beta = −0.49, *p* = 0.004, with age, hypertension and dyslipidemia configuring as other significant contributors) when adjusted for other CV risk factors (age, hypertension, disease duration, HbA1c, fasting glucose, cigarette habit, BMI, dyslipidemia) in a multiple regression model.

## Discussion

The main finding of our study was the significant and inverse correlation between the endothelial function, measured by FMD, and the left ventricular filling pressures, evaluated as E/e′ by transthoracic echocardiography. The higher FMD, the lower E/e′. Moreover, the FMD remained a significant predictor of E/e′ when embedded in a model with other cardiovascular risk factors classically associated with diastolic dysfunction. The diastolic dysfunction is common in diabetes, and partially attributable to the passive stiffness of the left ventricle due to the interstitial fibrosis that affects the myocardial tissue [11]. In fact, the chronic deposition of collagens fibers and AGEs in the myocardial fibers has been demonstrated in subjects with diabetes [12]. On the other hand, endothelial function has a fundamental role in the diastolic function of the left ventricle. The NO release from the coronary and endocardial endothelial cells hastens relaxation and enhances LV compliance [4, 13, 14]. In detail, NO released from the coronary endothelium facilitates myocardial relaxation via a cGMP-dependent reduction in myofilament Ca^2+^ sensitivity [15]. Therefore, NO modulates the myofilament responsiveness to Ca^2+^, thus helping the adaptation of the cardiac output to the pre-load changes [16]. In the comparison with controls, E/e′ was significantly higher in subjects with diabetes. We have also found statistically significant lower values of e′ in T2D compared to controls, and positive correlation between FMD and e′, both lateral and septal. The e′, an additional parameter for the assessment of the diastolic function [17], depends on LV relaxation and restoring forces [18]. Its reduction, both in septal and in lateral LV wall, confirms the presence of a subclinical diastolic dysfunction in T2D. The positive correlation we found between e′ and FMD supports the hypothesis that endothelial function contributes to regulate the active relaxation and diastolic distensibility [4]. We further observed that the deceleration time and the isovolumetric relaxation time were reduced in diabetes compared to controls. The mitral deceleration time and the IVRT could be reduced in case of elevated heart rate or when the left atrial pressure increases, the latter resulting in increased E/e′ ratio [18]. In addition, the higher LV mass and septum wall thickness observed in T2D suggest the presence of early signs of structural remodeling. For an appropriate interpretation of these findings, significantly different from individuals without diabetes but not fully abnormal, it must be underlining that T2D recruited had a quite short disease duration, a negative history of cardiovascular disease, no severe microvascular diabetes complications, and an acceptable glycemic control. We also observed a significantly low FMD in T2D, almost halved compared to controls. This despite the stimulus inducing NO-mediated vasodilation, SR_AUC 0-pd_, was significantly higher in T2D, suggesting an important dysfunction in the endothelium of these subjects. It is worth nothing that baseline diameter in T2D was significantly higher than controls, therefore possibly conditioning the FMD values. However, when absolute diameter change was compared, the difference between T2D and controls remained statistically significant. Many studies, conducted in pre-clinical models and in subjects with type 1 diabetes, explored and documented the simultaneous presence of endothelial and diastolic dysfunction [19–21]. Conversely, in type 2 diabetes, the relationship between endothelial and diastolic dysfunction was found only in people with the co-existence of diabetes and hypertension [22] or when diabetes was complicated by albuminuria [23]. Other studies found the association between FMD and left ventricular diastolic functional reserve but not in resting conditions [24], or only with endothelial-independent vasodilation [25]. Our study identified a significant and independent correlation between endothelial-dependent vasodilation and different parameters of diastolic dysfunction as E/e′ and both lateral and septal e′, reinforcing the concept that diabetes can also be defined as a cardio-vascular disease [26]. The cause-effect relationship between diastolic and endothelial dysfunction is complex, as the limit between reversible functional and structural damages are not well defined. However, the early characterization of individuals with diabetes and, therefore, the early intervention can prevent the development of diabetes myocardium remodeling [27, 28]. One of the limitations of our study was the lack of assessment of the global longitudinal strain (GLS). GLS has been demonstrated to be a sensitive tool to detect ventricular systolic dysfunction, even in a subclinical phase [29]. Lately, the strain has also been used to study the left atrium, whose stiffness is another feature suggestive of diastolic dysfunction [2]. Another limitation is that we studied the endothelial function not directly in the heart but at the brachial artery, in the peripheral vasculature. The reasons for this choice are the non-invasive nature of the technique and the demonstration that it is an adequate surrogate for the coronary endothelial function [30, 31].

## Conclusions

This observational study demonstrates a link between endothelial and diastolic dysfunction in subjects with type 2 diabetes, in absence of severe diabetes complications and cardiovascular diseases. Endothelial function contributes to the intricate process leading to diastolic dysfunction and, consequently, in the development of diabetic cardiomyopathy.

## Data Availability

The raw data supporting the conclusions of this article will be made available by the authors on request.
